# Supplementary material for: Molecular model of a bacterial flagellar motor in situ reveals a “parts-list” of protein adaptations to increase torque
Source: bioRxiv. 2024 Oct 9:2023.09.08.556779. Preprint. [Version 2] doi: 10.1101/2023.09.08.556779 (PMC11482838; doi:10.1101/2023.09.08.556779)
Supplement: Supplement 1 [file NIHPP2023.09.08.556779v2-supplement-1.pdf]

bioRxiv preprint doi: https://doi.org/10.1101/2023.09.08.556779; this version posted October 9, 2024. The copyright holder for this preprint (which was not certified by peer review) is the author/funder, who has granted bioRxiv a license to display the preprint in perpetuity. It is made available under aCC-BY 4.0 International license.

Supplemental table titles

Table S1: PflA and PflB pulldowns and mass spectrometry

Table S2: Structural components of the *Campylobacter jejuni* flagellar motor

Table S3: Antisera, strains, and recombinant DNA used in this study

(*Files provided separately in XLSX format*)

TABLE S1

PflA co-immunoprecipitation results

Accession	Protein	% Coverage	# PSMs	Ratio Abundance (D*pflA*/*pflA-FLAG*/D*pflA*/vector)
A0A0H3PAU5	PflA	95	979	2.87E+03
A0A0H3PI11	Cjj81176_1608,	60	13	6.72E+02
A0A0H3P9Z2	Cjj81176_0859,	22	17	2.39E+02
A0A0H3PBX6	MotB	39	11	1.58E+02
A0A0H3PIR6	Cjj81176_0166	56	32	1.37E+02
Q29W27	KpsD, capsular p	27	29	9.38E+01
A0A0H3PAU1	PflD	33	4	7.26E+01
A0A0H3PBN5	Cjj81176_0293,	39	8	6.85E+01
A0A0H3PGP7	FlgE, flagellar ho	58	47	6.70E+01
A0A0H3PCN0	Cjj81176_0127,	58	36	5.97E+01
A0A0H3PJ87	PflB	91	581	5.76E+01
A0A0H3PJ16	ModA, molybde	27	6	4.76E+01
A0A0H3PCR9	Cjj81176_1227,	29	8	4.49E+01
A0A0H3PIF6	FliL	35	6	4.45E+01
A1VYV6	Cbf2, putative p	60	63	4.04E+01
A0A0H3P9I9	YchF, ribosome-	19	8	3.86E+01
A0A0H3PHE2	Cjj81176_0792,	17	9	3.50E+01
A0A0H3PC06	Cjj81176_0400,	11	2	3.17E+01
A0A0H3PEG3	FliO	44	18	2.96E+01
A0A0H3PDV4	Cjj81176_1419,	44	17	2.96E+01

Accession	Protein	% Coverage	# PSMs	Abundance in D*pflA*/*pflA-FLAG* *
A0A0H3P9L7	Cjj81176_0128,	26	16	5.10E+06
A0A0H3PEV8	PbpA, penicillin-	20	14	4.15E+06
A0A0H3PE83	PflC	37	13	9.45E+06
A0A0H3P9U7	JlpA, surface-ex	26	10	3.12E+05
A0A0H3PIX5	Cjj81176_0480,	27	7	2.29E+06

A0A0H3PHT3	Cjj81176_1375,	27	6	1.20E+06
A0A0H3PHU2	Cjj81176_1517,	11	5	4.58E+05
A0A0H3P9C0	Cjj81176_1195,	19	4	4.32E+05
A0A0H3PDG0	Cjj81176_0851,	9	3	6.13E+05
A0A0H3PBC1	Cjj81176_0178,	5	3	5.43E+05
A0A0H3PD23	Cjj81176_1105.	15	3	1.30E+05
A0A0H3PAF3	Cjj81176_0231 ,	25	3	1.14E+06
A0A0H3PEX7	Cjj81176_0438,	15	3	2.44E+05
A0A0H3PAT4	Cjj81176_1652,	3	2	6.99E+05
Q0Q7H1	AtpE, ATP synth	31	2	3.65E+05
A0A0H3P986	Cjj81176_0968,	8	2	5.18E+05
A1W170	FlgI	9	2	3.68E+05
A0A0H3PD29	CobB, NAD-depe	3	2	8.70E+04
A0A0H3PBM4	Cjj81176_0677,	21	2	3.98E+05
A0A0H3PAG3	SdhC, succinate	6	2	5.73E+05
A0A0H3PBJ5	DsbD, thiol:disul	5	2	5.00E+04
A0A0H3PIZ5	Cjj81176_0157,	4	2	1.88E+05
A0A0H3P9K1	Cjj81176_0815,	4	2	1.88E+05
A0A0H3PA19	Cjj81176_0835,	4	2	1.88E+05

* These proteins were not detected by mass spectrometry in D*pflA* /vector. Thus, no abundance ratios can be calcualted.

PflB co-immunoprecipitation results

**Accession**	**Protein**	**% Coverage**	**# PSMs**	**Ratio Abundance (D*pflB*/*pflABFLAG*/D*pflB*/vector)**
A0A0H3PJ87	PflB	91	581	2.39E+03
A0A0H3PIR6	Cjj81176_0166,	56	32	4.38E+02
A0A0H3PEG3	FliO	44	18	1.02E+02
A0A0H3P9H3	DsbA, thiol:disul	38	12	3.92E+01
Q29W27	KpsD, capsular p	27	29	3.83E+01
A0A0H3P9J8	CjaC protein	18	6	3.11E+01
A0A0H3PDV4	Cjj81176_1419,	44	17	2.81E+01
A0A0H3PAU5	PflA	95	979	2.59E+01

A0A0H3PCT8	Cjj81176_1302,	17	21	2.42E+01
A0A0H3PIF6	FliL	35	6	2.28E+01
A0A0H3PAR8	MltG, endolytic	18	8	1.61E+01
A1VYV6	Cbf2, putative p	60	63	1.49E+01
A0A0H3P9T5	Cjj81176_1649,	13	11	1.31E+01
A0A0H3PCN0	Cjj81176_0127,	58	36	1.14E+01
A0A0H3PG98	CJJ81176_pVir0	40	23	1.11E+01
A0A0H3PI11	Cjj81176_1608,	60	13	1.08E+01
A0A0H3PHN8	Cjj81176_0836,	50	16	1.07E+01
A0A0H3PJ16	ModA, molybde	27	6	1.03E+01
A0A0H3P9F0	PglF, general gly	11	7	8.41E+00
A0A0H3PA52	HtrA, periplasmi	40	22	8.24E+00

**Accession**	**Protein**	**% Coverage**	**# PSMs**	**Abundance in D*pflA*/*pflA-FLAG* ***
A0A0H3P9U7	JlpA, surface-ex	26	10	3.15E+06
A0A0H3P9L7	Cjj81176_0128,	26	16	2.62E+06
A0A0H3PEV8	PbpA, penicillin-	20	14	2.06E+06
A0A0H3PA77	Cjj81176_0918,	24	3	5.03E+05
A0A0H3PIX5	Cjj81176_0480,	27	7	3.95E+05
A0A0H3PHU2	Cjj81176_1517,	11	5	3.72E+05
A0A0H3PAZ8	Cjj81176_0160,	6	1	3.50E+05
A0A0H3PAJ6	Cjj81176_0479,	10	5	2.88E+05
A0A0H3PAH4	Cjj81176_0565,	22	4	2.53E+05
A0A0H3PJB7	SdhB, succinate	5	5	2.39E+05
A0A0H3PHT3	Cjj81176_1375,	27	6	2.04E+05
A0A0H3PJH9	CtpA, carboxyl-t	9	3	1.64E+05
A0A0H3PAQ1	Cjj81176_0849,	2	1	1.57E+05
A0A0H3PC06	Cjj81176_0400,	11	2	1.50E+05
A0A0H3PJE1	Cjj81176_0214,	5	1	1.31E+05
A0A0H3P9Y0	Cjj81176_1228,	2	2	1.21E+05
A0A0H3P9H6	Cjj81176_0967,	5	1	9.57E+04

A0A0H3PBM4	Cjj81176_0677,	21	2	8.29E+04
A0A0H3PBT1	Cjj81176_0149,	2	1	8.24E+04
Q0Q7H1	AtpE, ATP synth	31	2	7.76E+04
A0A0H3PAA9	Cjj81176_1435,	10	5	7.16E+04
A0A0H3PBJ1	KpsE, capsular p	7	2	7.00E+04
A0A0H3P9Y9	Ldh, L-lactate de	5	1	6.99E+04
Q2M5Q6	Cjj81176_1318,	2	1	6.30E+04
A0A0H3PD23	Cjj81176_1105,	15	3	5.26E+04
A1W170	FlgI	9	2	5.02E+04
A0A0H3PE93	LolD, lipoprotein	9	2	4.89E+04
A0A0H3PBA0	CarA, carbamoy	2	1	4.70E+04
A0A0H3PBJ5	DsbC, thiol:disul	5	2	4.23E+04
Q0Q7I0	Cjj81176_1569,	9	4	3.86E+04
A0A0H3P994	Cjj81176_0144,	6	1	3.72E+04

* These proteins were not detected by mass spectrometry in D*pflA* /vector. Thus, no abundance ratios can be calcualted.

TABLE S2

**Protein**	**Accession number**	**Functional description**	**Color in figures**	Approx. resolution (d99) cite PMID 30198894	**Stoichiometry**	**Region modelled**	**Structural source**
MotB	A0A0H3P BX6	Stator unit component	Light pink	>10	2 (x17)	15-55 (TM) 68-247 (periplasmic)	From PMID 32931735 AlphaFold2
FliL	A0A0H3PI F6	MotB-associated periplasmic protein	Dark red	>10	4 (x17)	81-178	Homology modelfrom PMID 35046042
PflA	A0A0H3P AU5	Scaffold protein that recruits PflB	Light green	9.1	1 (x17)	16-788	AlphaFold2
PflB	A0A0H3P J87	Scaffold protein that recruits stator complexes by MotB	Dark green	>10	1 (x17)	138-820	AlphaFold2

PflC	A0A0D7VI M6	Scaffold protein that attaches the scaffold to the basak disk, makes up the medial disk	Magenta, teal	9.3 - 9.7	1 (x17)+ 6 (x17)	16-364	AlphaFold2
PflD	A0A0H3P AU1	Scaffold protein	Black	>10	1 (x17)	91-162	AlphaFold2
FlgP	A0A0H3P CP8	Makes up the basal disk, a large rigid OM-asociated brace	Purple	9.9	51	66-171	AlphaFold2

TABLE S3

REAGENT or RESOURCE	SOURCE	IDENTIFIER
Antibodies		
FlgP specific antisera	Ref[21]	FlgP specific antisera
Anti-GFP	Roche	#11814460001, RRID:AB_390913
Anti-FLAG	Sigma-Aldrich	#F1804-1MG, RRID:AB_262044
Anti-mouse IgG	GE Healthcare	#RPN4201
Anti-rabbit IgG	GE Healthcare	#RPN4301
Bacterial and virus strains		
C. jejuni 81-176 *rpsL*^{Sm} (Sm^R)	Ref[53]	*C. jejuni* DRH212
C. jejuni 81-176 Δ*flhG* Δ*flaAB*	This work	*C. jejuni* minicell-producing strain
C. jejuni 81-176 *pflA*$_{\Delta 18\text{-}168}$	This work	*C. jejuni* PflA truncation
C. jejuni NCTC11168 wildtype	This work	*C. jejuni* NCTC11168 WT CSS-0032
C. jejuni NCTC11168 Δ*cj1643*	This work	*C. jejuni* PflC deletion CSS-4087
C. jejuni NCTC11168 Δ*cj0892c*	This work	*C. jejuni* PflD deletion CSS-4081
C. jejuni NCTC11168 Cj1643-3xFLAG	This work	*C. jejuni* PflC-3xFLAG CSS-4720
C. jejuni NCTC11168 Δ*cj0892c* + *cj0892c-sfgfp*	This work	*C. jejuni* PflD-sfGFP CSS-4666
C. jejuni NCTC11168 Δ*cj0892c* + *cj0892c-sfgfp* , PflA-3xFLAG	This work	*C. jejuni* PflD-sfGFP, PflA-3xFLAG CSS-5714
C. jejuni NCTC11168 Δ*cj0892c* + *cj0892c-sfgfp* , PflB-3xFLAG	This work	*C. jejuni* PflD-sfGFP, PflB-3xFLAG CSS-5716
C. jejuni 81-176 Δ*flgQ / pDRH7476*	This work	*C. jejuni* FlgQ-mCherry DRH7516
E. coli DH5α	Lab stock	Cloning strain
E. coli BL21(DE3)	Lab stock	Protein expression strain
C. jejuni 81-176 *rpsL*^{Sm} Δ*pflA*	Ref[9]	*C. jejuni* DAR1124
C. jejuni 81-176 *rpsL* Δ*pflA/pDAR3417*	This work	*C. jejuni* DAR3447

C. jejuni 81-176 *rpsL* *ΔpflA/pDAR1604*	This work	*C. jejuni* DAR3477
C. jejuni 81-176 *rpsL*^{Sm} *ΔpflB*	Ref[9]	*C. jejuni* DAR981
C. jejuni 81-176 *rpsL* *ΔpflB/pDAR3414*	This work	*C. jejuni* DAR3451
C. jejuni 81-176 *rpsL* *ΔpflB/pDAR965*	This work	*C. jejuni* DAR3479
C. jejuni 81-176 *ΔflaA* *flaB*^{S397} *flhF*^{D321A}*ΔcheY*	This work	CCW-locked *C. jejuni* with subpolar short filaments for bead assay
C. jejuni 81-176 *rpsL* Δ*flgX/pDRH8743*	This work	DRH8754
Deposited data		
Raw single particle analysis movies	This work	EMPIAR-11580 (DOI: 10.6019/EMPIAR-10016)
Whole motor map	This work	EMD-16723
Lathed LP-ring focused refinement map	This work	EMD-16723 (additional volume)
Lathed C-ring focused refinement map	This work	EMD-16723 (additional volume)
Lathed MS-ring focused refinement map	This work	EMD-16723 (additional volume)
Focused periplasmic scaffold map	This work	EMD-16724
Δ*pflC* subtomogram average	This work	EMD-17415
Δ*pflD* subtomogram average	This work	EMD-17416
pflA$_{\Delta 16\text{-}168}$ subtomogram average	This work	EMD-17417
FlgQ-mCherry subtomogram average	This work	EMD-17419
C-ring subtomogram average	This work	EMD-19642
Recombinant DNA		

Source of *cat* cassette for chloramphenicol resistance	Ref[81]	pRY109
E. coli-C. jejuni shuttle vector	Ref[81]	pRY112
pRY112 with 76-bp fragment containing *cat* promoter with start codon and in- frame BamHI restriction site cloned into the XbaI and XmaI sites	This work	pDAR1003
pDAR1003 with DNA encoding *mcherry* and stop codon cloned in-frame with respect to the *cat* start codon and BamHI site into the XmaI and EcoRV sites	This work	pDAR1006
Fusion with *flgQ* from codon 2 to the penultimate codon cloned into the BamHI site of pDAR1006 to create a FlgQ-mCherry fusion	This work	FlgQ-mCherry plasmid pDRH7476
E. coli-C. jejuni shuttle vector	Ref[81]	pRY108
E. coli-C. jejuni shuttle vector containing cat promoter and start codon for expression of genes for complementation	Ref[82]	pECO102
E. coli-C. jejuni shuttle vector containing cat promoter and start codon followed by DNA encoding an in-frame N-terminal FLAG tag	Ref[78]	pDAR965
pRY108 with 206 base pair fragment containing flaA promoter and start codonwith in-frame SpeI site cloned into the XbaI and BamHI sites	This work	pDAR1425

E. coli-C. jejuni shuttle vector containing flaA promoter and start codon followed by DNA encoding an in-frame N-terminal FLAG tag	Ref[83]	pDAR1604
pECO102 with codon 2 to penultimate codon of pflB and an in-frame C-terminal FLAG epitope cloned into the BamHI site	This work	pDAR3414
pDAR1425 with codon 2 to penultimate codon of pflA and an in-frame C-terminal FLAG epitope cloned into the BamHI site	This work	pDAR3417
pECO102::*flgX-lysozyme*	This work	pDRH8743
pLIC-PflA	This work	Full-length PflA (16-788)
pLIC-PflAα	This work	PflA TPR regions (169-788)
pLIC-$PflA_N$	This work	PflA N-terminal half (16-454)
pLIC-PflB	This work	Soluble PflB (113-820)
pLIC-PflC	This work	Full-length PflC (17-364)
pLIC-$PflC_{\Delta 236-349}$	This work	PflC C-terminal truncation (17-235)
pLIC (Amp^R)	Franziska Sendker	Cloning vector backbone
